# Acyl post-translational modification of proteins by metabolites in cancer cells

**DOI:** 10.1038/s41420-025-02535-4

**Published:** 2025-05-21

**Authors:** Xudong Wang, Yining Guo, Yutian Fu, Chen Zhang, Weiwu Chen, Xinyu Tang, Yanlan Yu, Yicheng Chen, Guoqing Ding, Jie Zhang

**Affiliations:** 1 Department of Urology, Sir Run Run Shaw Hospital, School of Medicine, Zhejiang University, Hangzhou, China; 2 Department of General Surgery, Sir Run Run Shaw Hospital, School of Medicine, Zhejiang University, Hangzhou, China; 3 National Engineering Research Center of Innovation and Application of Minimally Invasive Instruments, Sir Run Run Shaw Hospital, School of Medicine, Zhejiang University, Hangzhou, China

**Keywords:** Cancer metabolism, Post-translational modifications

## Abstract

The relationship between metabolism and cancer is a major focus of current research, with an increasing number of studies highlighting the significant role of various metabolites in tumor cells, such as lactate, acetic acid, lysine, serine, tryptophan, palmitic acid, succinate, etc. These metabolites are involved in numerous biological processes within tumor cells, including transcription, translation, post-translational modification (PTM) of proteins, cell cycle regulation, and metabolism, thereby modulating tumor proliferation, migration, and drug resistance. Metabolite-mediated PTMs of proteins undoubtedly play a vital role in tumor cells, affecting both histones and non-histone proteins, covering modifications such as lactylation, crotonylation, acetylation, palmitoylation, and succinylation. Therefore, this review aims to elaborate on the abnormal levels of some major metabolites, related metabolic pathways, and the latest protein acyl PTMs they mediate in tumor cells, providing new insights for diagnosis and therapy in the field of oncology.

## Facts

- Cancer is closely linked to metabolic reprogramming.
- Key metabolites and their metabolic pathways are involved in various biological processes.
- Metabolites can affect the progression of tumors largely by acyl PTM.
- A variety of histones and non-histone proteins can undergo acyl PTMs.

## Open questions

- What is the mechanism by which metabolic reprogramming affects the progression of the tumor?
- What are the metabolic pathways of key metabolites that impact tumor progression?
- Which proteins influence tumor progression through acyl PTMs?
- Do different PTM types (e.g., lactylation vs acetylation) exhibit competition or synergy under the same metabolite context?
- How does the dynamic concentration gradient of metabolites in the tumor microenvironment influence the spatial distribution of PTMs?

## Introduction

Recent advancements in research have increasingly demonstrated that the progression of cancer is closely linked to metabolic reprogramming and abnormal levels of metabolites. These metabolites are involved in various metabolic pathways, including glucose metabolism, glutamine metabolism, lactate metabolism, pyruvate metabolism, ketone body and fatty acid metabolism, amino acid metabolism, etc [1]. Additionally, they can also maintain redox balance to meet the energy demands for tumor cells, which are significantly different from those of normal cells [2]. Metabolites are the products of synthesis or decomposition metabolism in cells, microorganisms, and other organisms, or substances ingested by them [3]. They can be distributed within tumor cells or in the tumor microenvironment [4]. Abnormal levels of metabolites in conjunction with mutations in cancer-related genes can not only lead to the activation of oncogenes, inactivation of tumor suppressor genes, but also result in aberrant functions, protein stability, localization, interactions, and activity within tumor cells [5]. Additionally, these abnormalities are intricately connected to the functions of various cells in the tumor microenvironment, such as macrophages, T cells, natural killer (NK) cells, endothelial cells, and fibroblasts [4, 6].

Recent studies have increasingly revealed a close relationship between abnormal levels of metabolites and the progression of cancer. Metabolites such as lactate, uridine, fatty acids, amino acids, polyamines, flavonoids, etc, exhibit heterogeneity across different tumors [7–10]. The concentration differences of metabolites in tumor cells and the tumor microenvironment can significantly affect the proliferation, migration, and drug resistance of tumor cells [1], as well as the immune response of immune cells in the microenvironment [11, 12]. In addition to their traditional roles in bioenergy and biosynthesis, recent studies have also uncovered some new pathways of influence, among which PTMs of proteins in tumor cells are a particularly crucial one.

Given that numerous PTMs of proteins have been reported before, in this review, we focus on the latest acyl PTMs of proteins mediated by metabolites in tumor cells, and pay attention to the latest advancements in this specific field. Our aim is to elucidate the entire process and importance of how changes in the concentration of specific metabolites mediate the progression of specific tumors through acyl PTMs of proteins, and provide new ideas and insights for the clinical diagnosis and treatment of tumors.

### Lactate and lactylation

Lactate, as a metabolic product of glycolysis, was once considered to be merely a harmful metabolic byproduct produced under hypoxic conditions, but an increasing number of studies have demonstrated that lactate plays a crucial role in the regulation of whole-body metabolism [13]. The Warburg effect indicates that regardless of the oxygen availability, the metabolic reprogramming in tumor cells will inhibit oxidative phosphorylation of mitochondria, so the utilization of glucose tends to be anaerobic glycolysis, rather than aerobic oxidation, which will lead to the accumulation of lactate [14]. A study in 2019 further revealed that lactate can promote the post-translational modification (PTM) of lysine residues in histones, that is, lactylation, thus mediating regulatory effects at the transcriptional level [15]. Therefore, lactate and lactylation are important biological factors that contribute to the initiation and progression of tumors.

**Fig. 1:**
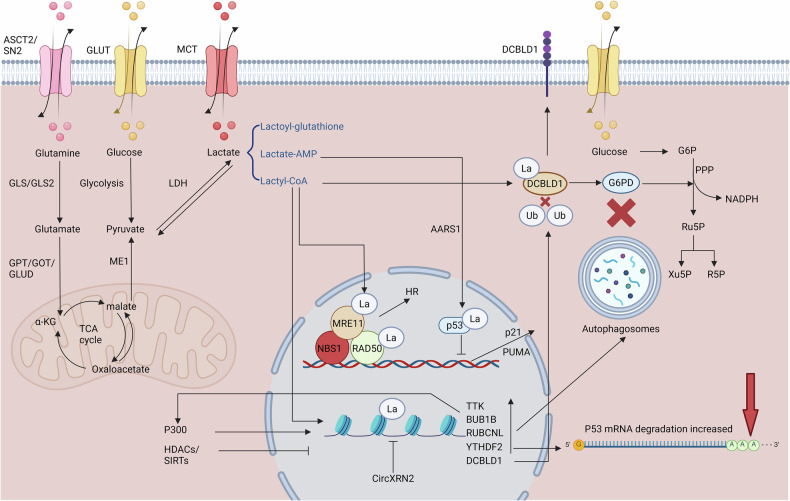
Metabolic pathways of lactate and protein lactylation in tumor cells. Lactate enters tumor cells via MCT receptors or is synthesized from glucose and glutamine metabolism. It mediates protein lactylation (La) through the formation of lactoyl-glutathione, lactyl-CoA, or lactate-AMP. Lactylation of MRE11 and NBS1 enhances MRN complex formation, facilitating HR-mediated DNA repair and chemoresistance. Lactylation of p53 reduces its tumor suppressor function by inhibiting its DNA binding and transcriptional activity. Oncogene DCBLD1 lactylation blocks its ubiquitination-dependent degradation, stabilizing G6PD and activating the PPP pathway to promote tumor progression. Additionally, histone H3K18 lactylation upregulates key genes like TTK, BUB1B, YTHDF2, DCBLD1, Wnt/β-catenin, NF-κB, and PD-L1, fostering tumor progression and drug resistance. Created with BioRender.com.

From a metabolic perspective, lactate is a byproduct of glycolysis. In the cytoplasm, glucose is catalyzed by a series of enzymes such as hexokinase, phosphofructokinase-1, and pyruvate kinase, to ultimately yield pyruvate. When the body is in a state of hypoxia, infection, intense exercise, etc, and the cell’s demand for ATP increases, pyruvate and NADH tends to be directly catalyzed by lactate dehydrogenase (LDH) to produce lactate, rather than entering the mitochondria to participate in the tricarboxylic acid cycle (TCA). Lactate can also serve as a major circulating carbohydrate fuel. Through the action of enzymes such as LDH, it can be reconverted into glucose via gluconeogenesis, thereby providing energy for the body [16]. At the same time, the catabolism of glutamine is also an important source of lactate in tumor cells [17]. Glutamine, regulated by c-MYC, is facilitated by type 2 amino acid transport protein (ASCT2) and sodium-coupled neutral amino acid transport protein 5 (SN2) to cross the cell membrane, enter the cytoplasm, and be converted into glutamate by glutaminase (GLS/GLS2). Subsequently, glutamate can be catalyzed by transaminases such as glutamate-oxaloacetate transaminase (GOT), glutamate-pyruvate transaminase (GPT), or glutamate dehydrogenase (GLUD), to produce α-ketoglutarate, which enters the TCA cycle. Within the TCA cycle, α-ketoglutarate will eventually generate oxaloacetate, which is converted to malate under the action of malate transaminases. Malate exits the mitochondria, and is subsequently converted to pyruvate and NADPH by malate dehydrogenase (ME1). Pyruvate can then serve as a crucial substrate for the production of lactate [18]. At the same time, lactate can also be transported into cells via monocarboxylate transport proteins (MCT1-4), facilitating a lactate shuttle mechanism between glycolytic cells and oxidative cells (Fig. 1) [19].

Lactate-induced protein lactylation generally manifests in two primary forms: lactyl-CoA and lactoyl-glutathione. Lactoyl-CoA is typically associated with enzymatic lactylation. processes, which can be generated by guanosine triphosphate (GTP)-specific SCS (GTPSCS) or acyl-CoA synthetase short-chain2 (ACSS2) in the nucleus [20, 21], while lactoyl-glutathione is closely linked to non-enzymatic lactylation, often occurring in proteins involved in glycolysis [22]. Additionally, recent research has demonstrated that in tumor cells, lactate and ATP can be catalyzed by alanyl-tRNA synthetase, specifically AARS1, to form lactate-AMP, which subsequently combines with the lysine residues of proteins, resulting in lactylation [23]. Classical acyltransferases (KAT family) and deacylases (HDAC and SIRT family) are believed to catalyze all lactylation modifications and delactylation processes, but several non-classical acyltransferases are also included, such as AARS1, AARS2 (Fig. 1) [23, 24]. Lactyltransferases, such as p300, CBP, and TIP60, can catalyze the transfer of the lactyl group from lactyl-CoA to the lysine residues of target proteins [25].

Lactylation modification has been proven to occur on a diverse array of proteins, encompassing both histones and non-histone proteins, and is involved in mediating the onset and progression of tumors. A multitude of non-histone proteins within tumor cells can undergo lactylation modification, thereby participating in multiple biological processes of cells, such as DNA damage response (DDR), transcriptional activity, pentose phosphate pathway (PPP), etc. Chen et al. identified that the key protein MRE11 in the MRN complex can undergo lactylation, mediated by acetyltransferase CBP. The lactylated MRE11 is more likely to bind to DNA, thereby promoting the phosphorylation of ATM, and ultimately facilitating the cutting and homologous recombination (HR) at the ends of DNA, mediating the chemoresistance of tumor cells. Administration of CBP or LDH inhibitors, or cell-penetrating peptides that can specifically block the lactylation of MRE11, can reduce the lactylation of MRE11, inhibit HR, and enhance the sensitivity of tumor cells to chemotherapeutic agents such as cisplatin, PARPi, etc [26]. Similarly, Chen et al. discovered that the lactylation of NBS1 at lysine 388 (K388) can promote the accumulation of the MRN complex and HR proteins at the site of DNA double-strand breaks (DSBs), thereby facilitating DNA damage repair, which is frequently associated with poor outcomes of tumor patients undergoing neoadjuvant chemotherapy [27]. Another study also elucidated that AARS1 can function as a lactate sensor, catalyzing the generation of lactate-AMP, and mediating the lactylation of p53, and the lactylation of p53 will inhibit its liquid-liquid phase separation, DNA binding capabilities, and ultimately lead to a decrease in transcriptional activity, fostering the onset and development of tumors. Elevated expression of AARS1 and lactylation of p53 are often associated with poor prognosis of patients, and administration of β-alanine to antagonize the lactylation of p53, coupled with doxorubicin to activate the transcriptional activity of p53, can significantly inhibit the proliferation of tumor cells in animal models [23]. In addition, Meng et al. revealed that DCBLD1, as an oncogene, can also undergo lactylation, and the lactylation of DCBLD1 can inhibit its ubiquitination, thereby stabilizing the DCBLD1 protein. Highly expressed DCBLD1 can impede the autophagic degradation of glucose-6-phosphate dehydrogenase (G6PD), thereby activating the pentose phosphate pathway (PPP) and promoting the progression and metastasis of cervical cancer. Inhibition of the activity of G6PD using 6-An can significantly suppress tumor growth and proliferation [28]. Moreover, a variety of proteins, including cGAS [24], PKM2 [29], METTL16 [30], and HMGB1 [31], have also been found to undergo lactylation modification (Fig. 1).

Similarly, lactylation also occurs extensively in histones of tumor cells. Li et al. discovered that lactate, produced through glycolysis in pancreatic ductal adenocarcinoma (PDAC), can promote H3K18la under the catalysis of P300, thereby enhancing the transcription of mitotic checkpoint regulatory factors TTK and BUB1B. This process ultimately leads to the increased expression of P300, thus forming a positive feedback loop and promoting the occurrence and development of tumors [32]. Another study also demonstrated that lactate, produced under hypoxic conditions in colorectal cancer (CRC), promotes the lactylation of H3K18, which in turn induces the transcription of autophagy enhancer protein RUBCNL, then promotes the maturation of autophagosomes, and mediates the recruitment of class III phosphatidylinositol 3-kinase(PI3K) complexes, thereby promoting tumor proliferation and mediating tumor resistance to bevacizumab [33]. Yu et al. found that histone H3K18la can also enhance the transcription of YTHDF2, and YTHDF2 can recognize m6A on PER1 and TP53 mRNA, and promote its degradation, thereby contributing to the development and progression of ocular melanoma [34]. Regarding the upstream mechanism of histone lactylation, Xie et al. reported that CircXRN2 can inhibit the degradation of LATS1, thereby activating the Hippo signaling pathway, inhibiting glycolysis and lactate production, subsequently suppressing H3K18la, and ultimately inhibiting the progression of bladder cancer mediated by histone lactylation [35]. Furthermore, emerging research has elucidated the significance of interaction between lactyl-CoA synthetase and lactyltransferase within the nucleus, which can facilitate the lactylation of histone H3 at lysine 18 (H3K18), such as ACSS2 and KAT2A, as well as GTPSCS and p300. This histone modification has been shown to upregulate key pathways and proteins, including Wnt/β-catenin, NF-κB, PD-L1, and GDF15, thereby contributing to immune escape and therapeutic resistance in brain tumors (Fig. 1) [20, 21].

In summary, tumor cells tend to convert glucose to lactate via aerobic glycolysis. However, as the malignancy progresses, due to difficulties in vascular and immune cell infiltration, more glucose may be converted to lactate through anaerobic glycolysis, leading to its accumulation and potentially promoting tumor progression further.

### Lysine and crotonylation

Lysine, as a branched-chain amino acid, can participate in various biological processes such as biosynthesis, energy provision, and maintenance of redox balance. Simultaneously, increasing evidence has indicated that lysine and its derivatives can participate in the metabolic reprogramming of tumors, mediate the occurrence and metastasis of tumors, and PTM is one of the key mechanisms involved [36].

**Fig. 2:**
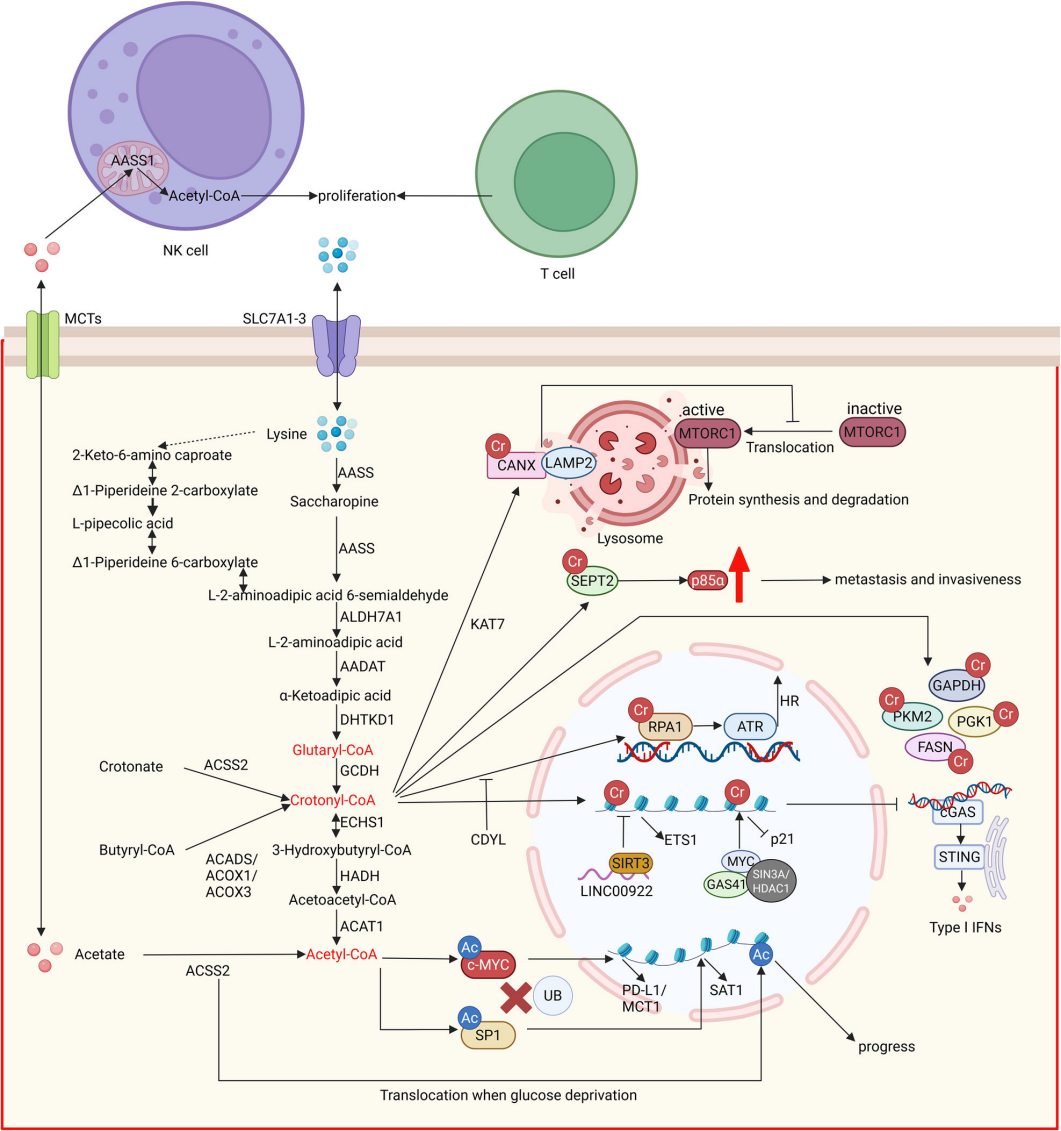
Metabolic pathways of lysine, acetate, and protein crotonylation, acetylation in tumor cells. Lysine imported via SLC7A1-3 is converted to crotonyl-CoA by enzymes like AASS and GCDH, facilitating protein crotonylation (Cr). Crotonylation of RPA1 enhances its binding to single-stranded DNA damage and HR proteins, promoting DNA repair and chemoresistance. Leucine deprivation induces CANX crotonylation, disrupting its lysosomal translocation and inhibiting MTORC1 signaling. SEPT2 crotonylation stabilizes p85α, driving metastasis and invasion. Histones H3 and H4 can also undergo crotonylation, upregulating ETS1, downregulating p21, and dsDNA/dsRNA, contributing to tumor progression. Acetate enters cells via MCT and is converted to acetyl-CoA by ACSS2. c-MYC acetylation (Ac) prevents its ubiquitination, boosting PD-L1 transcription and immune evasion. SP1 acetylation stabilizes itself, regulating tumor progression via polyamine metabolism. Histone can also undergo acetylation, and it is linked to brain tumor progression. In immune cells, mitochondrial ACSS1 can generate acetyl-CoA, promoting T and NK cell proliferation. Created with BioRender.com.

In tumor cells, lysine uptake is facilitated through membrane receptors such as SLC7A1, SLC7A2, and SLC7A3, with SLC7A2 being the predominant transporter [37]. The saccharophine pathway is considered to be the main pathway for the catabolism of lysine and yields two units of acetyl-CoA ultimately, but contemporary studies have increasingly focused on the intermediate products of this metabolic pathway. Specifically, in cells, lysine is sequentially catalyzed by enzymes including AASS, ALDH7A1, AADA, DHTKD1 to generate glutaryl-CoA, which is then catalyzed by crotonyl-CoA-producing enzyme glutaryl-CoA dehydrogenase (GCDH) to produce crotonyl-CoA, that is, the precursor for the crotonylation of histones and non-histone proteins [37]. At the same time, the produced crotonyl-CoA can also be hydrolyzed by crotonyl-CoA hydratase, enoyl-CoA hydratase short chain 1 (ECHS1), and ultimately converted to acetyl-CoA under the catalysis of HADH, ACAT1, and other enzymes [38]. Many tumor cells often exhibit high expression of GCDH and low expression of ECHS1, leading to the accumulation of crotonyl-CoA and promoting the crotonylation modification of the proteome (Fig. 2).

The precursor of crotonylation modification is crotonyl-CoA. Classical acyltransferases (KAT family) and deacylases (HDAC and SIRT family) are believed to catalyze all crotonylation and decrotonylation modifications. Crotonyltransferases, including p300, PCAF, and MOF, are capable of transferring the crotonyl group from crotonyl-CoA to the lysine residues of target proteins [39]. Additionally, YEATS2 can function as a histone-selective crotonyl transferase. Studies have shown that the lysine residues subject to crotonylation are often flanked by an overexpression of glutamic acid (E) at the −1 and +1 positions [39].

Crotonylation modification occurs in a variety of histones and non-histone proteins, playing a role in tumor onset and metastasis. Regarding non-histone proteins, Yu et al. discovered that RPA1 can undergo crotonylation at K88, K379, and K595, and the RPA1 modified by crotonylation is more likely to bind to damaged single-stranded DNA and HR proteins, thereby promoting DNA damage repair and the survival of tumor cells. Chromodomain Y-like (CDYL) can act as a hydrolase for crotonyl-CoA, inhibiting the crotonylation modification of proteins like RPA1 [40]. Another study revealed that deprivation of leucine can promote the crotonylation of CANX at K525, thereby mediating the lysosomal translocation of CANX. The translocation of CANX can interact with RRAG GTPases through LAMP2, and consequently, alter the activity of the MTORC1 signaling pathway. Inhibiting or genetically knocking out its acyltransferase KAT7 can make the MTORC1 pathway no longer sensitive to leucine deprivation [41]. Additionally, Zhang et al. found that the crotonylation of SEPT2 at K74 can promote the metastasis and recurrence of hepatocellular carcinoma (HCC) via the downstream SEPT2-K74cr-P85α-AKT pathway, leading to treatment failure. SIRT2 could decrotonylate SEPT2, and CBP, p300 could crotonylate SEPT2 [42]. Moreover, PGK1, PKM2, HK2, IDH1, ENO1, FASN, GAPDH, Vincullin, and numerous other non-histone proteins exhibit crotonylation modifications and are involved in various biological processes such as metabolism, autophagy, transcription, transport, and stem cell differentiation (Fig. 2) [43].

Similarly, histones in tumor cells can also undergo a large amount of crotonylation. Liao et al. found that LINC00922 can bind to deacylases SIRT3, preventing its combination with the promoter region of ETS1, thereby leading to an increased crotonylation of H3K27, promoting the transcription of ETS1, and ultimately mediating the metastasis of colorectal cancer [44]. In contrast, another study revealed the transcriptional inhibitory effect of H3K27 crotonylation. It was found that the oncogene MYC can recruit the GAS41/SIN3A-HDAC1 complex to promote H3K27cr, thereby mediating the transcriptional repression of cell cycle inhibitor protein p21, thus promoting colorectal cancer onset and progression [45]. In addition, Yuan et al. identified the crotonylation of H4K5, H4K8, H4K12, and its role in promoting the growth of glioblastoma. They also found that the reduction of histone crotonylation modification can promote the production of dsRNA and dsDNA in the cytoplasm, thereby activating the cGAS-STING pathway and enhancing type I interferon signal transmission, resulting in a tumor suppressor effect (Fig. 2) [37].

The association between amino acid metabolism and PTMs has been less explored before. As more aspects of amino acid catabolism are elucidated, the research of their connections with PTMs may be further expanded and revealed.

### Acetate and acetylation

Acetyl-CoA, as an important intermediate product in carbon metabolism, can participate in the TCA cycle, lipid and steroid synthesis, protein acetylation, and many other biological processes, thereby regulating energy generation, cell proliferation, gene transcription, etc. When tumor cells are in a stress environment, such as hypoxia or high glycolytic metabolism, glucose tends to be predominantly converted into lactate rather than acetyl-CoA due to the Warburg effect. Under these conditions, acetic acid becomes a crucial source of acetyl-CoA in tumor cells [46].

Monocarboxylate transporters (MCTs or SLC16) and sodium-coupled MCTs (SMCTs), with MCT1 being the primary transporter in tumor cells, are considered to be transport proteins for acetic acid [47]. The acyl-CoA synthetase short-chain family members (ACSSs) can convert short-chain fatty acids into short-chain acyl-CoA, and the acetic acid, transported into tumor cells, is mainly catalyzed by nuclear-cytoplasmic localized ACSS2, rather than mitochondrial-localized ACSS1, ACSS3, to produce acetyl-CoA [48]. Notably, ACSS1 can catalyze the production of acetyl-CoA from acetic acid in immune cells within the tumor microenvironment, thereby promoting the proliferation of T cells and NK cells and contributing to the clearance of tumor cells [48]. The precursor for acetylation modification is acetyl-CoA, and classical acyltransferases and deacetylases are believed to catalyze all acetylation modifications and deacetylation modifications, including non-classical acyltransferases such as dihydrolipoamide *S*-acetyltransferase (DLAT) at the same time (Fig. 2). Acetyltransferases transfer the acetyl group from acetyl-CoA to specific amino acid residues of target proteins. Based on the amino acid sites, this acetylation process can be classified into lysine acetylation, N-terminal acetylation, and O-acetylation [49].

Acetylation modification can occur on a variety of histones and non-histone proteins. For non-histone proteins, Wang et al. discovered that acetic acid can mediate the metabolic reprogramming of tumor cells and serve as a precursor to promote the acetylation of c-Myc. The acetylated c-Myc at Lys148 residue, mediated by DLAT, enhances the binding affinity to USP10. This interaction antagonizes ubiquitination-mediated degradation, thereby promoting c-Myc protein stability. Functioning as a transcription factor, the c-Myc upregulates the transcription of PD-L1, MCT1, glycolytic enzymes, and cell cycle accelerators. These molecular events collectively lead to suppressed T cell infiltration and enhanced tumor growth. The supplementation of acetate can promote the growth of lung tumors, while the limitation of acetate uptake can inhibit tumor immune escape and promote the efficacy of PD-L1 immunotherapy [47]. Similarly, another study identified that pancreatic cancer-associated fibroblasts (CAFs) can secrete acetic acid into the stroma of pancreatic cancer, which is mediated by ATP citrate lyase (ACLY). The acetic acid within the tumor environment can then be taken up by tumor cells and promote the acetylation of transcription factor SP1. The acetylation of SP1 at K19 increases its protein stability and transcriptional activity, affecting polyamine metabolism through the ACSS2–SP1–SAT1 axis, and promoting the occurrence and progression of tumors. The depletion of SP1 or pharmacologic inhibition of the ACSS2–SP1–SAT1 axis could diminish the tumor burden [50]. In addition, Hu et al. also found the association between gut flora and tumors, noting that acetic acid secreted by L. reuteri can induce acetylation at the K30 position of the transcription factor Sox13 in type 3 innate lymphoid cells (ILC3). The acetylation of Sox13 reduces its expression at the mRNA level, subsequently decreasing the production of IL-17A, and ultimately enhancing the anti-tumor effect of ILC3 in the liver (Fig. 2) [51].

At the same time, acetylation of histones mediated by acetic acid is also prevalent. For example, Li et al. found that when cells are exposed to an environment of low sugar and other nutrient deficiencies, acetyl-CoA synthase ACSS2 will translocate to the nucleus and catalyze the production of acetyl-CoA from acetic acid, thereby mediating the acetylation of histone H3. The acetylation of histones in specific promoter regions will promote the transcription of related genes, thereby facilitating the development of brain tumors (Fig. 2) [52].

From the perspective of the metabolic pathway of lysine, there exists a significant association between crotonyl-CoA and acetyl-CoA. Whether they exert mutual antagonism or mutual promotion, as well as the regulatory mechanisms involved, remains unclear, which is still of urgent need for further research.

### Palmitic acid (PA) and palmitoylation

In addition to contributing to obesity and other metabolic diseases, a high-fat diet is increasingly considered to be closely related to the onset and progression of tumors. As one of the primary sources of dietary oil, palm oil is a significant contributor to obesity, and an increasing number of studies indicate that it can affect the nutritional components of the tumor microenvironment and induce the progression and metastasis of tumors. As the principal component of palm oil, the saturated fatty acid, PA, along with its active form, palmitoyl-CoA, plays a crucial role in the progression of tumors through mediating protein PTMs and signal transduction pathways [53].

**Fig. 3:**
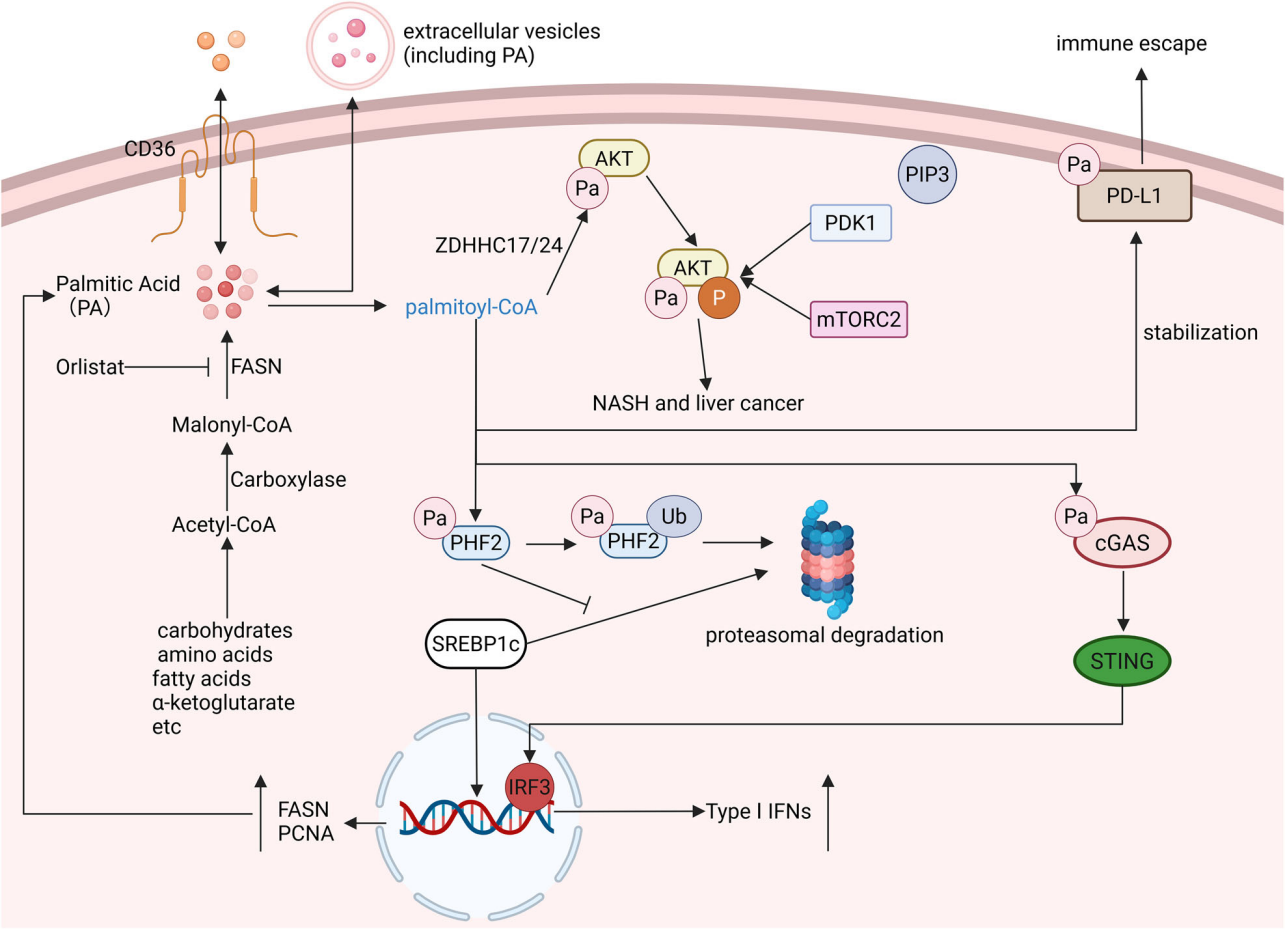
Metabolic pathways of palmitate and protein palmitoylation in tumor cells. Palmitate enters cells via CD36 or is synthesized intracellularly through DNL. Palmitoylation (Pa) of AKT facilitates its membrane localization, independent of PIP3, thereby activating downstream signaling pathways. Palmitoylation of PHF2 promotes its ubiquitination and degradation, leading to the stability of downstream substrates like SREBP1c, which enhances transcription of FASN and PCNA. Additionally, palmitoylation of PD-L1 enhances its stability, while that of cGAS enhances its activity, bolstering innate immune responses. Created with BioRender.com.

The uptake of PA by cells often requires the mediation of fatty acid transport protein CD36 [54], and it can also be encapsulated within extracellular vesicles or particles (EVPs) to enter and exit cells via endocytosis and exocytosis [55]. Excessive intake of dietary fat often results in elevated levels of PA in the body, and it can also be synthesized endogenously in the body through de novo lipogenesis (DNL). DNL is a conserved biological pathway that can utilize carbohydrates, amino acids, fatty acids, α-ketoglutarate, and other substrates to produce PA through the catalysis of acetyl-CoA carboxylase, fatty acid synthase, etc [56]. Fatty acid synthase FasA mainly synthesizes oleic acid and PA, while FasB predominantly synthesizes PA (Fig. 3) [57].

Once PA enters the cell, it is converted into palmitoyl-CoA through the action of long-chain acyl-CoA synthetases (ACSL) [58]. Within cells, PA mediates protein palmitoylation as palmitoyl-CoA. This process is catalyzed by palmitoyl transferases zinc finger DHHC-type containing (ZDHHC) protein family (ZDHHC1–9 and ZDHHC11–24) and demodifying enzymes Acyl-protein thioesterases (APT1/2), palmitoyl protein thioesterases (PPT1/2), or alpha/beta hydrolase domain-containing protein 17A/B/C (ABHD17A/B/C) [59]. Palmitoylation is a reversible lipid modification where palmitate is covalently attached to cysteine residues through a thioester bond, which can significantly increase the hydrophobicity of proteins, thereby affecting their affinity for membrane structures, their membrane localization, protein-protein interactions, and signal transduction of the protein [60, 61].

Numerous studies have shown that various non-histone proteins undergo aberrant palmitoylation in tumor cells. For instance, Bu et al. discovered that PA derived from high-fat diets or synthesized aberrantly can promote the palmitoylation of AKT at C77 and C224, thereby facilitating the membrane localization of AKT independent of PIP3. This process is mediated by ZDHHC17/24 and promotes the phosphorylation and activation of AKT, partly by inhibiting the formation of inactive polymer. The phosphorylated AKT subsequently activates the downstream signaling pathways, promoting cell proliferation and migration, and mediating the occurrence of liver cancer. Administration of anti-obesity drugs such as orlistat or other specific penetrating peptides has been shown to effectively inhibit tumor progression [62]. In addition, Jeong et al. found that high levels of PA in HCC can promote the palmitoylation of PHF2, an E3 ubiquitin ligase, leading to its ubiquitination-dependent degradation. Consequently, as its substrate, the lipid synthesis transcription factor SREBP1c, will correspondingly increase, thereby promoting the positive feedback loop of lipid generation, including PA, and advancing the progression of HCC [63]. Furthermore, palmitoylation is closely associated with the efficacy of tumor immunotherapy. Specifically, ZDHHC9-mediated palmitoylation of PD-L1 at Cys272 enhances its protein stability, which subsequently suppresses T cell activation and facilitates tumor immune escape. In parallel, the palmitoylation of cGAS at Cys 404/405 promotes its functional activation through two distinct mechanisms: (1) augmenting cytoplasmic double-stranded DNA (dsDNA) sensing capacity, and (2) facilitating cGAS dimerization—a prerequisite conformational change for enzymatic activation. These coordinated molecular events ultimately potentiate the cGAS-STING signaling cascade, thereby amplifying innate immune responses. Both PD-L1 and cGAS can be palmitoylated by ZDHHC9, and cGAS can be depalmitoylated by LYPLAL1. Therefore, targeting the palmitoylation of PD-L1 and the depalmitoylation of cGAS represents a potential novel strategy to enhance the clinical effectiveness of anti-tumor immunotherapy [64, 65]. Furthermore, a diverse array of proteins, including β-catenin [66], GSDMD [60], NLRP3 [67], NRAS [68], and STING [69], have been demonstrated to undergo palmitoylation (Fig. 3).

Currently, there are still relatively few research articles focusing on the palmitoylation modification of histones in tumor cells. Han et al. discovered that LPCAT1 can promote the palmitoylation of histone H4, leading to an increase in mRNA synthesis, and thereby mediating the progression of castration-resistant prostate cancer (CRPC) [70]. In addition, another study discovered that PA can significantly alter the histone H3K4me3 levels at the promoters of the cellular genome through the stable deposition of the methyltransferase Set1A (a member of the MLL/COMPASS family) [53]. This alteration subsequently affects the transcriptional levels of numerous genes. Transcription factor binding analysis revealed that the enrichment of the transcription factor EGR2 at the promoter regions can promote the expression of a large number of nerve-related genes, such as the glial-inducing peptide galanin (GAL). Ultimately, it stimulates Schwann cells within the tumor to secrete a pro-regenerative extracellular matrix, mediating the prometastatic memory of the tumor [71]. Given that PA serves as a crucial substrate for palmitoylation, its impact on histone H3K4me3 may imply that there are numerous underexplored interrelationships between histone palmitoylation and methylation. These potential connections warrant further investigation to elucidate the intricate regulatory mechanisms underlying chromatin dynamics and gene expression.

## Succinate and succinylation

As the sole metabolite directly linking the TCA cycle with the mitochondrial respiratory chain, succinate’s multifaceted roles are progressively being elucidated, with evidence highlighting its pivotal functions in pro-inflammatory processes, ischemia-reperfusion injury, and metabolic signal transduction [72]. Research into succinate and its mediated succinylation in tumors is rapidly advancing.

Succinate, a key intermediate metabolite of the TCA cycle in the mitochondrial matrix, accumulates under conditions such as intense physical exertion, ischemia-reperfusion, or poisoning, where the mitochondrial respiratory chain is inhibited. The accumulation of NADH leads to a reduction in the coenzyme Q pool, and coupled with the malate–aspartate shuttle, a surplus of fumarate accumulates and is then converted to succinate by the reverse catalysis of succinate dehydrogenase (SDH) [73, 74]. The accumulated succinate can be transported into the cytoplasm via SLC25A10 to exert its functions [72]. Intracellular succinate can also be exported to the extracellular space and enter the circulatory system, such as in skeletal muscle cells, where succinate can be secreted into the extracellular milieu by MCT1 and bind to the succinate receptor-1 (SUCNR-1) on non-myofibrillar resident cells, activating downstream signaling pathways like ERK1/2 and PI3K-AKT [75, 76]. Additionally, extracellular succinate can be reuptake into cells, such as in brown adipose tissue under cold conditions, where it is taken up from the peripheral circulation and transported into mitochondria via SLC25A10 to participate in UCP1-dependent thermogenesis [77]. Moreover, α-ketoglutarate generated from glutamine catabolism within the cell can also enter the TCA cycle to produce succinate, which is subsequently converted back to fumarate by SDH.

Succinate can covalently mediate the succinylation of protein lysine residues in the form of succinyl-CoA, either enzymatically or non-enzymatically. Succinyl-CoA is generated under the catalysis of succinyl-coenzyme A ligase (SUCL), which consists of the α-subunit encoded by SUCLG1 and the β-subunit encoded by SUCLA2, formed from ATP or SUCLG2, formed from GTP [78]. Currently, the reported succinyltransferases and desuccinylases remain limited. The main succinyltransferases include KAT2A with the α-ketoglutarate dehydrogenase (α-KGDH) complex, histone acetyltransferase 1 (HAT1), CPT1A, OXCT1 [79–81], and classic acyltransferases such as p300 and CBP. Desuccinylases primarily encompass SIRT5 and SIRT7 [82], as well as the HDAC family, with HDAC1/2/3 primarily mediating the desuccinylation of histones [83].

In non-histone proteins, Ma et al. identified that OXCT1, an enzyme catalyzing ketone body oxidation, can act as a succinyltransferase, catalyzing the succinylation of the tumor suppressor protein LACTB at K284. The succinylation of LACTB suppresses its own proteolytic activity, leading to an increase in its substrates, such as mitochondrial enzyme phosphatidylserine decarboxylase (PISD), lysophosphatidylethanolamines (LPEs), and phosphatidylethanolamines (PEs) in mitochondria, thereby increasing mitochondrial membrane potential and mitochondrial respiration, enhancing mitochondrial function, and promoting the progression of HCC [81]. Furthermore, Hu et al. revealed that succinylation of SUCLG2 at K93 can inhibit TRIM21-mediated ubiquitination, thereby enhancing the stability of SUCLG2. This modification subsequently reduces the succinylation levels of several mitochondrial proteins, including GAPDH, ME2, IDH2, MDH2, and ACOT9. By modulating the stability or function of these proteins, SUCLG2 succinylation at K93 ultimately promotes the progression of lung adenocarcinoma [78]. Similarly, another study revealed the succinylation of the classic tumor suppressor protein p53 at K120, with SIRT5 functioning as its desuccinylase. The succinylation of p53 inhibits its function and transcriptional activity, which subsequently results in the suppression of p53 target gene expression. Consequently, this leads to the failure of cell arrest or cell apoptosis, and promotes cellular DNA damage and tumorigenesis [84]. Additionally, Shi et al. discovered that LncRNA GLTC can antagonize the binding of SIRT5 with LDHA, thereby promoting the succinylation of LDHA. This modification enhances its enzymatic activity, leading to increased aerobic glycolysis and metabolic reprogramming in tumor cells, which in turn promotes tumor progression [85]. Moreover, various proteins, such as GLS, FBN1, POLRMT, and Keap1, can also undergo succinylation modifications [86–89].

Concurrently, histones can undergo succinylation modifications. Shahidian et al. found that succinylation modification of histone H3 at K122 in the promoter region can promote gene transcription and increase nucleosome stability [90]. Li and Yang et al. also arrived at similar conclusions and revealed the correlation between histone succinylation and tumor progression [80, 83].

Due to the Warburg effect, tumor cells tend to generate lactate from glucose rather than channeling it into the TCA cycle, which further promotes the accumulation of succinate. Therefore, lactylation and succinylation may synergistically act to facilitate tumor progression.

## Regulatory networks of certain PTMs

As intermediate products of cellular metabolism, the complex regulatory network formed by the interactions and interconversions of metabolites is termed the metabolome [91]. Given that the sources and destinations of metabolites are intricately intertwined, it is inevitable that metabolites mediating acyl PTMs in tumor cells will generate potential crosstalk [92]. As previously mentioned, the catabolism of lysine can produce intermediates such as glutaryl-CoA and crotonyl-CoA, with the latter being converted to acetyl-CoA ultimately by enzymes like ECHS1 [37]. Similarly, the oxidation of fatty acids in mitochondria, including PA, can also generate crotonyl-CoA. In mitochondria, succinyl-CoA can be produced from propionyl-CoA [93], and it can also provide CoA to acetoacetate to form acetoacetyl-CoA, which is subsequently converted to two molecules of acetyl-CoA by mitochondrial thiolase [94]. Moreover, the production of many acyl-CoAs is regulated by the same enzymes, such as ACSS2 and ACSL, and the complex regulatory mechanisms among them are in urgent need of further detailed and precise investigation.

**Fig. 4:**
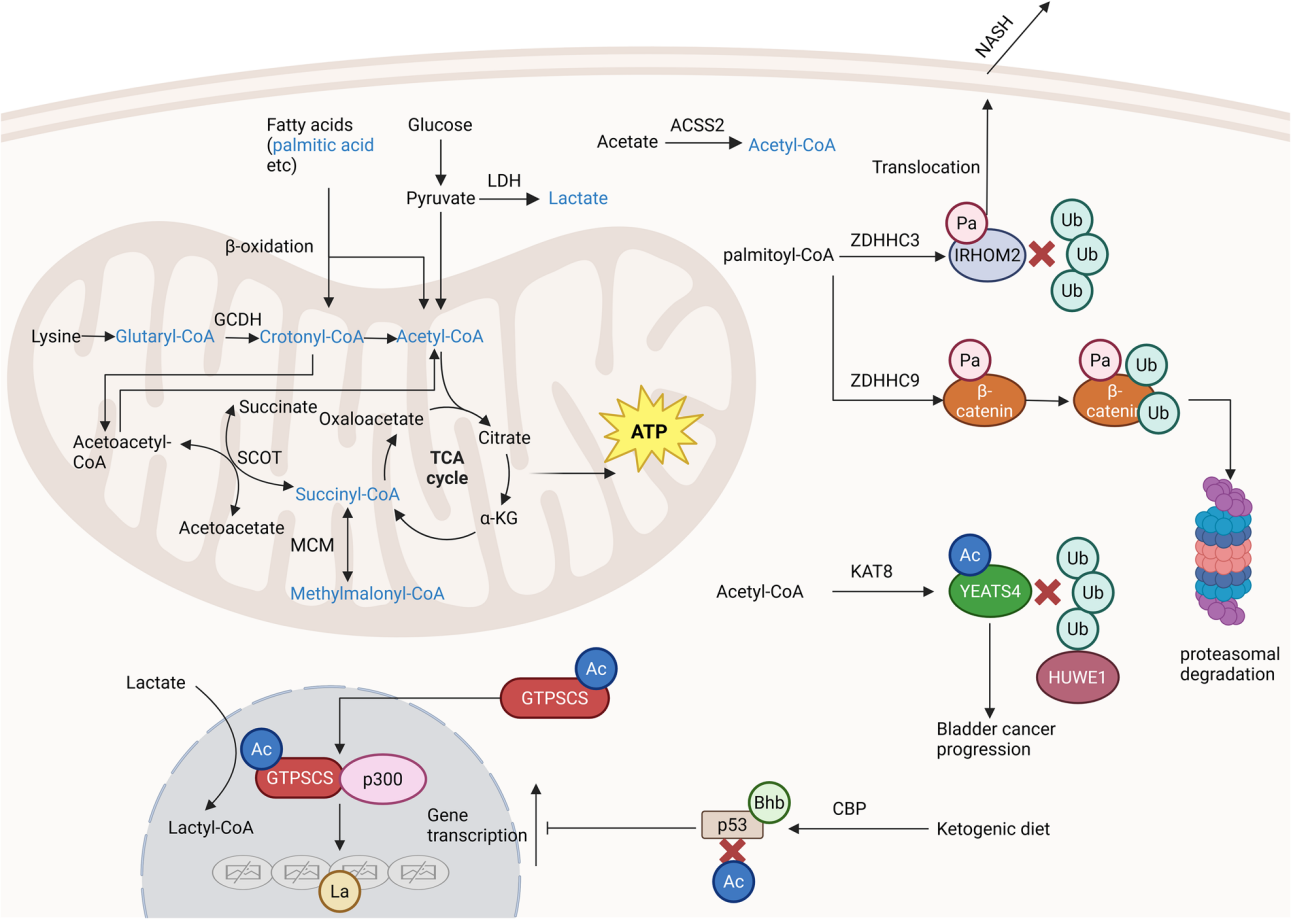
The regulatory networks of certain PTMs. Lysine catabolism produces glutaryl-CoA and crotonyl-CoA, which can be converted to acetyl-CoA by enzymes such as ECHS1. Fatty acids like PA generate crotonyl-CoA during mitochondrial oxidation. In mitochondria, succinyl-CoA can be derived from propionyl-CoA and can provide CoA for acetoacetate to ultimately produce acetyl-CoA. GTPSCS, when acetylated on its G2 subunit, can interact with p300 to mediate histone lactylation. Palmitoylation of IRHOM2 inhibits its ubiquitination and degradation, promoting its membrane translocation and contributing to NASH progression. Acetylation of YEATS4 antagonizes its ubiquitination and degradation mediated by HUWE1, facilitating bladder cancer progression. Conversely, palmitoylation of β-catenin promotes its ubiquitination and degradation, protecting against renal fibrosis. Additionally, β-hydroxybutyrylation (Bhb) of p53 inhibits its acetylation and suppresses its activity, thereby promoting tumor progression. Created with BioRender.com.

Given the interplay of precursor metabolites in metabolic pathways and the fact that multiple PTMs can occur on a single protein, these modifications can exert synergistic or antagonistic effects on protein function. For instance, Liu et al. reported that GTPSCS can translocate to the nucleus and interact with p300 to promote histone lactylation, a process that depends on the acetylation of the G2 subunit of GTPSCS [95]. Additionally, Xu et al. found that ZDHHC3 mediates the palmitoylation of IRHOM2 at C476, thereby inhibiting TRIM31-mediated ubiquitination and degradation. The stabilization and membrane translocation of IRHOM2 mediated by palmitoylation can eventually accelerate the progression of non-alcoholic steatohepatitis (NASH) [96]. Similarly, Xie et al. demonstrated that KAT8 mediates the acetylation of YEATS4, antagonizing its interaction with the E3 ubiquitin ligase HUWE1 and subsequent ubiquitination and degradation, thus promoting bladder cancer progression [97]. In contrast, the ZDHHC9-mediated palmitoylation of β-catenin promotes its ubiquitination and degradation, thereby protecting against renal fibrosis [66]. Moreover, the activity of p53 can be regulated by various PTMs, including lactylation, acetylation, succinylation, UFMylation, and β-hydroxybutyrylation. Among these, CBP-mediated β-hydroxybutyrylation can inhibit p53 acetylation, thereby suppressing p53 activity and promoting tumor progression (Fig. 4) [98].

Thus, not only do acylations interact with each other, but they also have close associations with other modifications such as ubiquitination, phosphorylation, and ubiquitin-like modifications. Many of these modifications are regulated by the same enzymes; for example, CBP can mediate both the β-hydroxybutyrylation of p53 and the lactylation of MRE11 [26, 98]. Accurate identification of the complex network of interactions among PTMs and how tumors coordinate these PTMs to act on different proteins to promote their progression is of critical importance.

## Discussion

PTMs is a biological process in which small-molecule substrate, such as acetyl-CoA, crotonyl-CoA, butyryl-CoA, phosphate, etc, is covalently bound to a specific amino acid residue of proteins in a reversible manner. To date, more than 450 forms of protein PTMs have been identified, and they interact with each other to form an essential regulatory network, which is an important switch for adjusting the functional state of cells [99]. Research on PTMs derived from metabolites in tumor cells and their function is currently developing rapidly. The latest acyl PTMs mediated by metabolites in tumor cells described in this review are listed in Table 1. In this review, we elaborated on several abnormal levels of metabolites, metabolic pathways, and PTMs they mediate in tumor cells. They can affect the survival, proliferation, metastasis, recurrence, drug resistance, and other functions of tumor cells through altering the function, activity, subcellular localization, protein-protein interactions, protein stability, other modification levels of non-histone proteins, and modifying histones to change gene expression, chromatin accessibility, transcriptional activity, all of which are closely related to the initiation and progression of tumors.

**Table 1:** PTMs by metabolites in cancer.

Name of modification	Metabolite	Residue modified	Protein targets	References
Lactylation	Lactate	Lysine	MRE11	[24]
			NBS1	[25]
			p53	[22]
			DCBLD1	[26]
			cGAS, PKM2, METTL16, HMGB1	[23, 27–29]
			Histone H3	[30–33]
Crotonylation	Lysine	Lysine	RPA1	[38]
			CANX	[39]
			SEPT2	[40]
			PGK1, PKM2, GAPDH, HK2, IDH1, ENO1, FASN, GAPDH, Vincullin, etc.	[41]
			Histone H3, H4	[35, 42, 43]
Acetylation	Acetate	Lysine	c-MYC	[45]
			SP1	[47]
			SOX13	[48]
			Histone H3	[49]
Palmitoylation	PA	Cysteine	AKT	[58]
			PHF2	[59]
			cGAS	[61]
			PD-L1	[60]
			β-catenin, GSDMD, NLRP3, NRAS, STING	[56, 62–65]
Succinylation	Succinate	Lysine	LACTB	[76]
			SUCLG2	[73]
			P53	[79]
			LDHA	[80]
			GAPDH, ME2, IDH2, MDH2, ACOT9, GLS, FBN1, POLRMT, KEAP1	[73, 81–84]
			Histone H3	[75, 78, 85]

It is believed that in the next few years, further research on how abnormal levels of metabolites affect the functional state of tumor cells through protein PTMs will gradually emerge. At the same time, these studies offer novel insights into the clinical diagnosis, treatment, and prognostic assessment of tumors. Traditional methods such as liquid chromatography-mass spectrometry (LC-MS) and gas chromatography-mass spectrometry (GC-MS), which integrate chromatography and mass spectrometry, are currently the mainstream techniques for detecting metabolites in tumors, but they face limitations in terms of low detection efficiency for metabolites with strong polarity, isomerism, and low ionization efficiency. Chromatography based on chemical derivatization offers a more efficient solution to these challenges. Its underlying principle is to connect the specific functional groups of metabolites, needed to be analyzed, with chemical derivatization reagents to enhance their stability and ionization efficiency, making them more suitable for chromatographic analysis [100].

Simultaneously, the aberration in metabolites and their modifications in tumors also offer new targets and pathways for us to treat tumors. The utilization of drugs targeting key enzymes in metabolic pathways of metabolites, or enzymes involved in metabolite modifications and demodifications, or knocking out related genes at the genomic level, is considered to be able to effectively inhibit the proliferation, metastasis, and chemoresistance of tumors. For instance, Chen et al. demonstrated that the lactylation of NBS1 can promote the formation of the MRN complex and homologous recombination-mediated DNA repair, thereby mediating the chemoresistance of tumors. Administration of stripentol, an LDHA inhibitor, or genetically knocking out LDHA can effectively inhibit the K388 lactylation of NBS1 and enhance the sensitivity of tumors to chemotherapy [27]. In addition, the development of cell-penetrating peptides targeting the inhibition of specific modified proteins or small molecule compounds antagonizing related modifications may also emerge as effective tumor treatment strategies.

More importantly, these studies point out that reasonable dietary intake and metabolic homeostasis in the body may prevent the onset and progression of tumors. For instance, Bu et al. found that adhering to a PA-restrictive diet can effectively inhibit the palmitoylation of AKT, thereby antagonizing the development of liver cancer [62].

However, the field of acyl PTMs continues to face numerous challenges and limitations. First, covalently attached PTMs often exist at sub-stoichiometric levels, representing only a small fraction of the entire proteome. The low abundance of these modifications poses a significant challenge for comprehensive PTM proteomics analysis [101]. Second, the sheer number of PTMs is vast and continually expanding, with new modifications such as alkylation [102] and vitcylation [103], being recently reported. A single protein can harbor multiple PTMs, and one PTM can often influence another, a phenomenon known as PTM crosstalk [104]. Understanding these complex mechanisms and regulatory networks is highly intricate, as most current studies have primarily focused on the effects of individual PTMs on single proteins. Additionally, developing targeted therapies based on metabolite-mediated protein PTMs remains a major challenge. Targeting metabolic enzymes or acyltransferases often suffers from poor specificity and significant side effects. Although there have been some advancements to target specific proteins, such as the lactyl-resistant knock-in system utilized by Li et al. to activate cGAS and prevent innate immune evasion in mice [24], the clinical efficacy and generalizability of such approaches are still uncertain.

We look forward to further revealing the mechanisms by which metabolites promote the progression of tumors via various types of PTMs, further clarifying the mutual relationship between metabolism and tumors, and utilizing them for the diagnosis, treatment, and prevention of tumors in the future.

## Data Availability

Not applicable.
